# Sex-related biological variation and mental toughness in soccer: a 2D:4D digit-ratio study of Type a, Type B and Type C classification across playing positions

**DOI:** 10.3389/fnbeh.2026.1934922

**Published:** 2026-08-25

**Authors:** Hamza Kaya Besler, Gulferi M. Yildirim, Sinan Canan, Sinan Seyhan, Görkem Açar, Muhammed Fatih Bilici, Uğur Sönmezoğlu

**Affiliations:** 1 Department of Sport Management, Faculty of Sports Sciences, Pamukkale University, Denizli, Türkiye; 2 Department of Neuroscience, Uskudar University, Istanbul, Türkiye; 3 Acikbeyin Education and Consultancy, Istanbul, Türkiye; 4 Department of Coaching Education, Faculty of Sport Sciences, Manisa Celal Bayar University, Manisa, Türkiye; 5 Faculty of Sport Sciences, Istanbul Gelisim University, Istanbul, Türkiye; 6 Faculty of Sport Sciences, Bitlis Eren University, Bitlis, Türkiye; 7 Department of Sport Management, Faculty of Sports Sciences, Pamukkale University, Denizli, Türkiye

**Keywords:** 2D:4D digit ratio, mental toughness, playing position, prenatal androgen exposure, sex-related biological variation, SMTQ, soccer, Type A/Type B/Type C classification

## Abstract

**Background:**

Sex-related variation in psychological traits is usually studied as a categorical male–female contrast, which conflates a grouping variable with the continuous biology underlying it. The second-to-fourth digit ratio (2D:4D) provides a non-invasive marker of the prenatal androgen environment, but its relationship with mental toughness and playing position in soccer remains poorly understood. This study examined the associations between 2D:4D-derived classification, playing position, sex and mental toughness, testing whether mental toughness tracks a continuous sex-related biological index rather than sex category alone.

**Methods:**

In a cross-sectional design, 270 licensed amateur soccer players (241 men, 29 women; aged 13–35 years) from Denizli, Türkiye, were assessed. Mental toughness was measured with the Sports Mental Toughness Questionnaire (SMTQ). Digit lengths were measured directly on both hands with a calibrated digital caliper, and the two-hand mean ratio assigned each player to one of three operational categories at a cut-off of unity (Type A < 1.00, Type C = 1.00, Type B > 1.00). Data were analysed with one-way and two-way ANOVA with Bonferroni *post-hoc* comparisons.

**Results:**

Type B players reported higher mental toughness (*M* = 47.36) than Type A (43.85) and Type C (44.24) players, *F*_(2, 267)_ = 6.70, *p* = 0.002, η^2^ = 0.048. Defenders (45.34) scored higher than goalkeepers (41.13; *p* = 0.021), *F*_(3, 266)_ = 3.00, *p* = 0.031, η^2^ = 0.033. In the two-way model classification remained significant, *F*_(2, 258)_ = 6.10, *p* = 0.003, whereas position, *F*_(3, 258)_ = 2.22, *p* = 0.086, and the interaction, *F*_(6, 258)_ = 0.81, *p* = 0.560, did not. Male players scored higher than female players, t(268) = 1.99, *p* = 0.047.

**Conclusion:**

Mental toughness varied with 2D:4D-derived classification and playing position, and the classification effect ran counter to a simple masculinization account, since the less androgenized Type B profile scored highest. A sex difference was also present, but classification and sex operated in opposing directions and neither was reducible to the other. Types A, B and C are operational classifications, not literal brain categories, and should be interpreted with caution.

## 1 Introduction

In the contemporary game, soccer challenges athletes physically and mentally alike. Whether professional or amateur, soccer is not merely a physical contest played on the pitch: talent, physical and mental training, financial status, management, stress regulation, sport psychology, and training science all shape performance (Carling et al., 2007; Diment, 2014; Surujlal, 2016). Although these factors are widely acknowledged, the neuroscientific underpinnings of such parameters remain debated within soccer research (Fink et al., 2019). When a prospective player is evaluated by a coach, talent, physical condition, and, to a lesser extent, psychological readiness are typically considered (Williams et al., 2020). Talent and physical condition can be observed and measured in numerous ways, yet quantifying neurophysiological tendencies is neither standardized nor straightforward (Kashima, 2000). Neurophysiological predispositions can, to some degree, be inferred from body parameters such as the 2D:4D ratio, which is thought to reflect prenatal, androgen-dependent organizational effects on the developing brain and may offer an indirect window onto how neural circuitry is configured before birth. Although the brain retains plasticity across the lifespan (Lustig et al., 2009), the developmental trajectory of any given brain may be constrained by its early, prenatally influenced wiring.

The ratio between the length of the index finger (2D) and the ring finger (4D) is termed the 2D:4D ratio (Manning et al., 2014). Converging evidence from animal (Brown et al., 2002; Manno, 2008; Auger et al., 2013) and human studies (Berenbaum et al., 2009; Manning et al., 2014) indicates that greater prenatal testosterone and lower estrogen exposure during the first trimester is associated with a lower 2D:4D ratio. On average, males exhibit lower 2D:4D ratios than females (Manning et al., 2000, 2014; van Honk et al., 2011). Nevertheless, the evidence linking 2D:4D to specific psychological traits is neither uniform nor fully consistent, and effect sizes are typically small (Hönekopp and Watson, 2010); results should therefore be interpreted as probabilistic rather than deterministic. A lower 2D:4D ratio has been associated with higher athletic performance and more assertive behavior, more so in males than in females (Hönekopp and Watson, 2010; Manning et al., 2017), and with higher scores for aggression and sensation-seeking in both sexes (Hampson et al., 2008). Assertiveness and high sport performance have, in turn, been linked to higher mental toughness (Bull et al., 2005; Gucciardi et al., 2017; Vaughan et al., 2018).

Framing this question in terms of sex-related biological variation, rather than athletic performance alone, matters for three reasons. First, 2D:4D is one of the very few non-invasive markers of the prenatal androgen environment that can be obtained in field settings, and prenatal androgen exposure is a principal mechanism through which sex-related variation in neural and behavioral phenotypes is thought to arise (Berenbaum et al., 2009; Manning et al., 2014). Second, sex-related variation in psychological traits is typically reported as a between-sex difference, which conflates a categorical grouping variable with the continuous biology assumed to underlie it; using a graded index that varies both within and between the sexes allows the two to be separated empirically. Third, sport remains a domain in which categorical assumptions about sex and psychological robustness are particularly entrenched, so evidence bearing on whether mental toughness tracks a continuous sex-related biological index rather than sex category itself carries both theoretical and applied weight. The present study is therefore positioned primarily as an examination of sex-related biological variation in a psychological trait, with playing position included as a contextual factor.

Positional demands are central to soccer, and each position requires a distinct physical and psychological profile (Bloomfield et al., 2007; Schumacher et al., 2018). A central defender may need to be mentally and physically robust to neutralize opponents, whereas a striker must be fast and creative to score. As a multitasking game, soccer requires players to attend to many cues simultaneously or, at other moments, to narrow attention onto a single target, depending on the tactical situation.

Building on this work, some authors have proposed a tripartite classification (Type A, Type B, Type C), described as reflecting prenatally organized, 2D:4D-linked differences in hemispheric functioning (Manning et al., 2000; Karaismailoglu, 2020). Throughout the present article these three labels are used as neutral, operational descriptors of digit-ratio profile; the terms male brain and female brain, and their variants, are deliberately avoided. In the present study we adopt this taxonomy as an organizing heuristic while explicitly acknowledging its limitations: strict left-brain/right-brain dichotomies and binary “male-brain/female-brain” models are increasingly contested, and large-scale imaging studies show that human brains are better described as mosaics of features than as two discrete sexual categories (Joel et al., 2015; Ingalhalikar et al., 2014; Eliot et al., 2021). We therefore treat the classification as a 2D:4D-derived proxy for prenatal androgenization, and not as a literal claim about categorically sexed brains.

Direct empirical evidence linking the 2D:4D ratio to this tripartite classification is, however, limited, and this should be stated plainly. The typology derives largely from applied neuroscience accounts (Manning et al., 2000; Canan, 2019; Karaismailoglu, 2020) in which relative digit length is treated as an accessible correlate of the prenatal androgen environment that shapes early neural organization; it does not derive from studies that have validated three discrete categories against neuroimaging or neuropsychological criteria. Peer-reviewed research has overwhelmingly treated 2D:4D as a continuous predictor of specific outcomes such as aggression, dominance, spatial ability or athletic performance (Hampson et al., 2008; Hönekopp and Watson, 2010; Perciavalle et al., 2013; Mailhos et al., 2016), and studies that operationalize it as Type A, Type B and Type C categories are correspondingly scarce. Accordingly, the present study does not assume that these categories index distinct neural systems. They are used as a transparent and reproducible way of partitioning a continuous, sex-related biological index at its natural cut-point of unity, and the empirical adequacy of the classification itself is treated as an open question that the present data help to inform rather than as a premise.

Within this framework, Type A–the more androgenized profile, in which the fourth digit exceeds the second–is described as result-oriented, analytic and single-focus, prioritizing solutions and outcomes over detail (Karaismailoglu, 2020). Type B–the less androgenized profile, in which the second digit exceeds the fourth–is described as multitasking-oriented, able to attend to several cues at once and to process fine detail. These descriptions are reported here as the claims of the source framework rather than as established findings, and the categories are referred to by their neutral labels throughout. Type C reflects a more balanced profile in which the hemispheres are engaged comparatively equally and which may, according to this model, be more amenable to training (Karaismailoglu, 2020).

**FIGURE 1:**
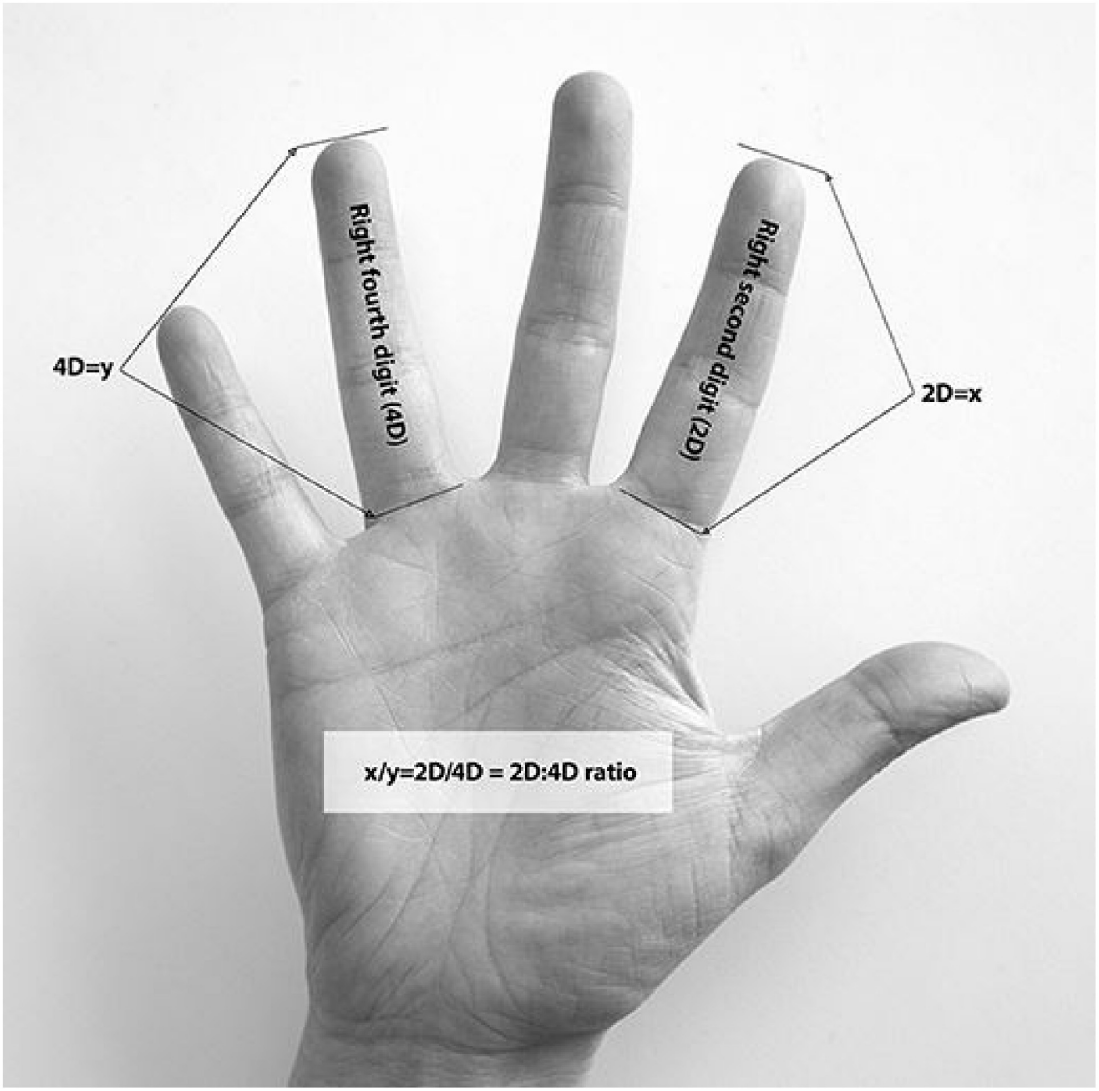
Measurement and calculation of the second-to-fourth digit (2D:4D) ratio (adapted from Eklund et al., 2020).

Historically, the 2D:4D ratio has been recognized as a sexually dimorphic trait (Baker, 1888; George, 1930), with lower mean values in males than in females (Phelps, 1952). More recent work reframes this dimorphism as a marker of prenatal testosterone exposure rather than of biological sex *per se* (Manning et al., 2000; Karaismailoglu, 2020). Accordingly, the 2D:4D ratio is best understood as one accessible, low-cost anthropometric proxy of prenatal hormonal milieu that may be informative when combined with psychological assessment, rather than as a stand-alone diagnostic of cognitive style. Figure 1 illustrates the measurement and calculation of the 2D:4D ratio (Eklund et al., 2020).

Soccer is a complex, multifaceted game that draws heavily on cognitive and psychological resources. Whereas prior research has largely focused on performance and aggression in relation to 2D:4D (Perciavalle et al., 2013; Bilgiç et al., 2016; Islam and Kundu, 2020), comparatively little work has examined how 2D:4D-derived categories relate jointly to mental toughness and playing position, and less still has framed the question explicitly in terms of sex-related biological variation. Mental toughness–an athlete’s capacity to remain determined, focused, confident, and in control under pressure– is a robust correlate of high performance (Clough et al., 2002; Jones et al., 2007; Gucciardi et al., 2017; Crust and Clough, 2011). Addressing this gap, the present study integrates a neuropsychological perspective with applied soccer research to test whether mental toughness varies with 2D:4D-derived classification and with positional role.

Two research questions guided the study:

Research Question 1. To what extent do mental toughness levels differ among soccer players assigned to the Type A, Type B and Type C categories on the basis of their 2D:4D ratio, and in which direction?

Research Question 2. To what extent do mental toughness levels differ according to playing position, and to what extent does playing position qualify the association between 2D:4D-derived classification and mental toughness?

The corresponding hypotheses were:

H1. Soccer players in the Type B category show higher mental toughness than players in the Type A or Type C categories.

H2. Mental toughness levels differ according to playing position.

## 2 Materials and methods

### 2.1 Study design

**FIGURE 2:**
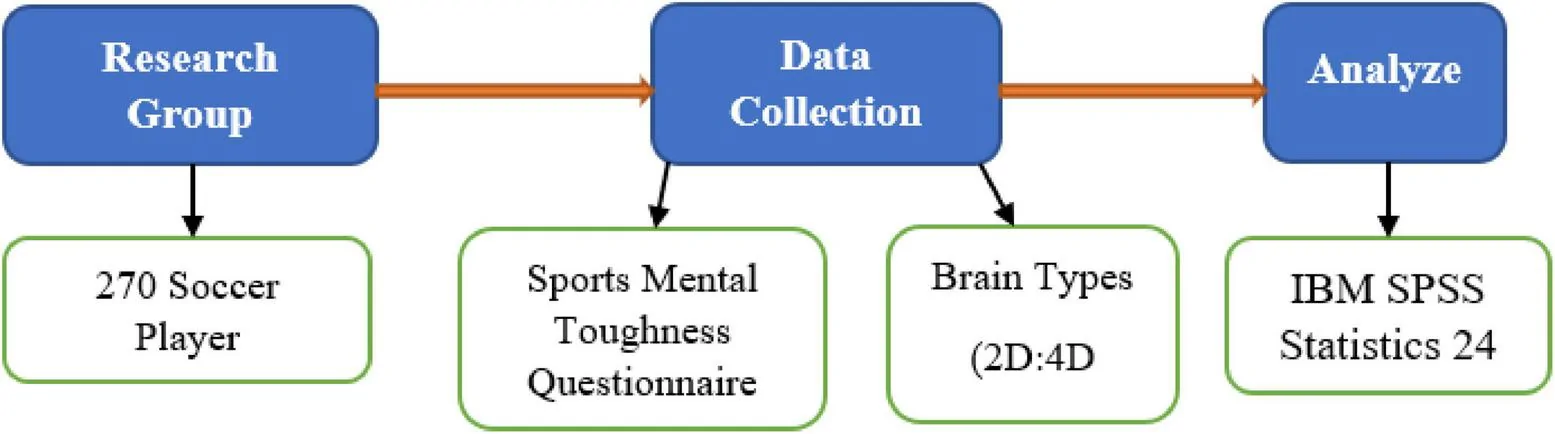
Research model.

A cross-sectional, quantitative survey design was used to examine the associations between 2D:4D-derived classification (Type A, Type B, Type C), playing position, and mental toughness in amateur soccer players (Figure 2).

An *a priori* power analysis was conducted using G*Power 3.1 to determine the minimum sample size (Faul et al., 2007). For an independent-samples *t*-test (two-tailed) with a medium effect size (*d* = 0.50; Cohen, 1988), an alpha level of 0.05, and a statistical power of 0.80, the analysis indicated that a minimum of 64 participants per group was required, yielding a total sample size of *N* = 128 (actual power = 0.80, critical *t* = 1.98, df = 126).

### 2.2 Participants

Purposive, volunteer sampling was used to recruit 270 licensed amateur soccer players from the Denizli region of Türkiye. Players competed in the Super Amateur League and the Amateur First Division. The sample comprised 241 men (89.3%) and 29 women (10.7%), with a mean age of 20.85 years. Inclusion criteria were a current amateur license, active competition during the 2022–2023 season, and provision of informed consent; players with hand or finger injuries or deformities that could affect digit measurement were excluded. The study protocol was reviewed and approved by the Social and Human Sciences Scientific Research and Publication Ethics Committee of Pamukkale University (approval date: 27/03/2023; decision number: 68282350/2023/07), and all participants provided written informed consent in accordance with the Declaration of Helsinki.

Age and sample composition. Age was recorded in bands, and the sample spanned adolescence to adulthood: 13–17 years (*n* = 84, 31.1%), 18–21 years (*n* = 84, 31.1%), 22–27 years (*n* = 90, 33.3%) and 28 years or older (*n* = 12, 4.4%). Approximately one third of the players were therefore below 18 years of age at the time of measurement. Participants were not stratified by chronological age, and we acknowledge directly that a substantial proportion of the sample had not completed skeletal maturation, since final adult stature and its segmental components can continue to develop in men into the mid-twenties. This is unlikely to compromise the classification applied here, and the reason is worth stating precisely. The variable of interest is not absolute digit length but the ratio between two digits of the same hand. This ratio is established prenatally under androgenic influence and thereafter remains broadly stable, because subsequent longitudinal growth acts on the second and fourth digits proportionally rather than altering the relationship between them, provided that no injury, deformity or pathology of the hand is present–conditions that were explicit exclusion criteria in this study. Residual growth therefore constrains the interpretation of absolute anthropometric measurements, but not of the ratio itself. The breadth of the age range is nonetheless reported as a limitation, since psychological maturation over adolescence may itself contribute to variation in mental toughness. Female players were retained rather than excluded. Excluding them would have increased the anthropometric homogeneity of the sample, but at the cost of the study’s central aim, which was to determine whether mental toughness tracks a continuous, sex-related biological index rather than sex category alone; a male-only sample cannot address that question. Retention also preserves the representativeness of a sample drawn from a licensed amateur population in which women’s football, although expanding rapidly in Türkiye, still constitutes a small minority of registered players. The consequences of the resulting sex imbalance for statistical inference are set out in the Data Analysis section, in the Results, and in the Limitations.

### 2.3 Measures

*Mental toughness.* Mental toughness was assessed with the Sports Mental Toughness Questionnaire (SMTQ; Sheard et al., 2009), in the Turkish version validated by Altıntaş and Koruç (2017). The SMTQ comprises 14 items rated on a 4-point Likert scale across three subdimensions (confidence, constancy, control); items 2, 4, 7, 8, 9, and 10 are reverse-scored. Total scores range from 14 to 56, with higher scores indicating greater mental toughness. Internal consistency in the present sample was acceptable (Cronbach’s α > 0.70; Davidshofer and Murphy, 2005).

Digit measurement (2D:4D). Digit lengths were measured directly on the participants’ hands–not from photocopies, photographs or scans–using a digital caliper with a resolution of 0.01 mm, which was checked against a reference standard before each measurement session. On both hands, the second (index) and fourth (ring) digits were measured on the ventral surface from the basal crease (the proximal flexion crease at the base of the digit) to the fingertip. During measurement, participants placed both hands flat on a horizontal surface at approximately elbow height, with the fingers naturally extended, fully relaxed, and neither flexed nor abducted; the caliper was applied without compressing the soft tissue. All measurements were performed by two of the authors, both trained in an identical anthropometric protocol, and every digit of every participant was measured twice. Where a discrepancy was observed between the two readings, a third measurement was obtained and the closest consistent values were retained. All measurements were taken under standardized conditions with the same calibrated instrument and the same protocol for every participant, in order to minimize both measurement error and observer-related error. It is acknowledged that direct caliper measurement, although widely used and practicable in field settings, is less precise than computer-assisted analysis of hand scans (Allaway et al., 2009; Zhao et al., 2013); formal inter- and intra-rater reliability coefficients were not computed, and this is stated as a limitation. Immediately after measurement, participants completed the study questionnaires under the supervision of the same researchers.

Operational classification (Type A, Type B, Type C). The 2D:4D ratio was computed separately for each hand as the length of the second digit divided by the length of the fourth digit. The arithmetic mean of the right- and left-hand ratios was then used for classification; no single hand was analysed in isolation, and hands were not classified separately. Classification followed the cut-off of unity used in the framework of Manning et al. (2000) and Karaismailoglu (2020). Participants whose mean fourth-digit length exceeded their mean second-digit length–that is, a mean 2D:4D ratio below 1.00–were assigned to Type A. Participants whose mean second-digit length exceeded their mean fourth-digit length–a mean 2D:4D ratio above 1.00–were assigned to Type B. Participants whose mean second- and fourth-digit lengths were equal–a mean 2D:4D ratio of exactly 1.00–were assigned to Type C. The three categories are therefore mutually exclusive and exhaustive by construction, and occupy non-overlapping intervals of the 2D:4D distribution (see Table 5). Throughout this article, Type A, Type B and Type C are used as neutral operational labels for these digit-ratio profiles. They are not literal brain categories, and no claim is made that they correspond to discrete neural systems.

### 2.4 Procedure

Club managers were informed and granted permission prior to data collection. Of the 270 players, 170 were invited to the Conference Hall of the Faculty of Sports Sciences at Pamukkale University, and 100 were visited at their club facilities. All players completed the SMTQ face-to-face under the supervision of the researcher, and 2D:4D measurements were taken on the same occasion.

### 2.5 Data analysis

Data were analysed using IBM SPSS Statistics 23. Normality of the study variables was assessed via skewness and kurtosis, and internal consistency was evaluated with Cronbach’s α. One-way analysis of variance (ANOVA) was used to compare mental toughness across classification categories and across playing positions, with Bonferroni-corrected pairwise comparisons for significant omnibus effects (George and Mallery, 2016). Because single-factor analyses cannot reveal whether the two factors act independently, a two-way (3 × 4) factorial ANOVA was additionally conducted with 2D:4D-derived classification (Type A, Type B, Type C) and playing position (defense, midfield, striker, goalkeeper) as fixed factors, together with their interaction term, so that the independent and joint contributions of the two factors could be evaluated simultaneously. A three-factor model additionally incorporating training experience was considered but was not retained: crossing three classification levels, four positions and four experience bands yields 48 cells for 270 players, and the resulting cell frequencies–particularly for goalkeepers (*n* = 23) and for players with 15 or more years of experience (*n* = 21)–were too sparse to support stable estimation of the higher-order interactions. Training experience is therefore reported descriptively, and adequately powered multifactorial designs are recommended in the Future Directions section. Effect sizes (eta-squared, η^2^) accompany each ANOVA to aid interpretation and comparison with future studies (Cohen, 2013), and exact F values and degrees of freedom are reported throughout. Statistical significance was set at *p* < 0.05.

Analytical treatment of biological sex. Both male (*n* = 241, 89.3%) and female (*n* = 29, 10.7%) players were recruited, and biological sex was treated as a descriptive characteristic of the sample rather than as a factor in the inferential models. Three considerations motivated this decision, and each is stated explicitly here because it bears directly on how the findings should be read. First, the research question concerned sex-related biological variation as indexed by a continuous, prenatally determined marker, not the categorical contrast between men and women; classification was therefore applied at the individual level irrespective of sex, so that players of both sexes are represented within each of the three categories. Second, the sex distribution of the sample was markedly unbalanced, reflecting the present composition of licensed amateur football in the region. Entering sex as a further factor alongside classification and playing position would have produced cells containing very few female players and, in several position-by-category combinations, none at all; the resulting estimates would have been unstable and the interaction terms uninterpretable. Third, because mean 2D:4D differs between the sexes, sex and classification are not statistically independent in this design, so treating them as separate crossed factors would have partitioned overlapping variance. Sex is consequently reported descriptively, and the resulting inability to test sex as a moderator is acknowledged explicitly in the Limitations.

## 3 Results

All study variables were approximately normally distributed, with skewness and kurtosis values within the ± 1.5 threshold, and internal consistency exceeded the 0.70 criterion (Davidshofer and Murphy, 2005). The sample of 270 players had a mean age of 20.85 years and comprised 89.3% men and 10.7% women. Regarding education, 53.0% had completed high school and 47.0% were university students or graduates. Playing experience was distributed as follows: 1–3 years, 20.0%; 4–7 years, 48.9%; 8–14 years, 23.3%; and 15+ years, 7.8% (Table 1).

**TABLE 1:** Demographic characteristics of the soccer players (*N* = 270).

Variable	Category	*n*	%
Age (years)	Mean = 20.85	270	–
Gender	Male	241	89.3
	Female	29	10.7
Education	High school	143	53.0
	University	127	47.0
Playing experience	1–3 years	54	20.0
	4–7 years	132	48.9
	8–14 years	63	23.3
	15+ years	21	7.8

Descriptive statistics for mental toughness by 2D:4D-derived classification and playing position are presented in Table 2. The overall mean SMTQ score was 44.64. Type A was the most common category (53.7%; mean = 43.85), followed by Type C (26.7%; mean = 44.24) and Type B (19.6%; mean = 47.36). By position, players were distributed across defense (34.4%), midfield (31.1%), striker (25.9%), and goalkeeper (8.5%); goalkeepers recorded the lowest mean mental toughness.

**TABLE 2:** Distribution and mean mental toughness by 2D:4D-derived classification and playing position.

Variable / Category	*n*	%	SMTQ mean
Classification–Type A	145	53.7	43.85
Classification–Type B	53	19.6	47.36
Classification–Type C	72	26.7	44.24
Position–Defense	93	34.4	45.34
Position–Midfield	84	31.1	44.95
Position–Striker	70	25.9	44.49
Position–Goalkeeper	23	8.5	41.13
Total	270	100	44.64

One-way ANOVA revealed that players assigned to Type B had significantly higher mental toughness (*M* = 47.36, SD = 5.87) than players assigned to Type A (*M* = 43.85, SD = 6.12) or Type C (*M* = 44.24, SD = 6.13), *F*_(2, 267)_ = 6.70, *p* = 0.002, η^2^ = 0.048. Bonferroni *post-hoc* comparisons confirmed that Type B exceeded both Type A (*p* = 0.001) and Type C (*p* = 0.015), whereas Type A and Type C did not differ significantly (Table 3).

**TABLE 3:** One-way ANOVA of mental toughness by 2D:4D-derived classification (Bonferroni *post-hoc*).

Scale / Classification	Mean	*p*	Bonferroni
SMTQ–Type B	47.36	–	B > A, B > C
SMTQ–Type A	43.85	0.001	
SMTQ–Type C	44.24	0.015	

*p* < 0.05.

One-way ANOVA showed a significant effect of playing position on mental toughness, *F*_(3, 266)_ = 3.00, *p* = 0.031, η^2^ = 0.033. Goalkeepers scored significantly lower (*M* = 41.13, SD = 6.02) than defenders (*M* = 45.34, SD = 6.46; *p* = 0.021). No other pairwise position comparisons reached significance, although the goalkeeper–midfielder contrast approached the threshold (*p* = 0.052; Table 4).

**TABLE 4:** One-way ANOVA of mental toughness by playing position (Bonferroni *post-hoc*).

Scale / Position	Mean	*p*	Bonferroni
SMTQ–Goalkeeper	41.13	–	GK < Defense
SMTQ–Defense	45.34	0.021	
SMTQ–Midfield	44.95	0.052	
SMTQ–Striker	44.49	0.142	

*p* < 0.05.

The composition of the three classification categories is reported in Table 5, together with the digit-length criterion and the corresponding 2D:4D interval that defines each category. Because classification was determined at the point of measurement by the position of the two-hand mean ratio relative to unity, the categories occupy mutually exclusive and non-overlapping intervals of the 2D:4D distribution by construction: Type A below 1.00, Type C exactly 1.00, and Type B above 1.00. Type A was the most frequent category, accounting for slightly more than half of the sample, which is consistent with the male predominance of the sample and with the lower mean 2D:4D values typically reported for men. We note as a limitation that the continuous 2D:4D values underlying this classification were not retained in the analytic dataset, so category means, standard deviations and observed minimum–maximum values cannot be reported here; future work in this laboratory will archive the continuous ratios alongside the categorical assignment.

**TABLE 5:** Composition of the 2D:4D-derived classification categories and their defining intervals (*N* = 270).

Category	Digit-length criterion (two-hand mean)	Defining 2D:4D interval	*n*	%
Type A	Fourth digit longer than second digit	2D:4D < 1.00	145	53.7
Type B	Second digit longer than fourth digit	2D:4D > 1.00	53	19.6
Type C	Second and fourth digits equal	2D:4D = 1.00	72	26.7
Total	–	–	270	100.0

2D:4D = length of the second digit divided by the length of the fourth digit. Classification was based on the arithmetic mean of the right- and left-hand ratios. Categories are mutually exclusive and exhaustive by construction. Continuous 2D:4D values were not retained in the analytic dataset; see the limitations.

A two-way (3 × 4) factorial ANOVA examining 2D:4D-derived classification and playing position simultaneously reproduced the pattern obtained in the single-factor analyses. The main effect of classification on mental toughness remained significant, *F*_(2, 258)_ = 6.10, *p* = 0.003, whereas the main effect of playing position did not reach significance once classification was accounted for, *F*_(3, 258)_ = 2.22, *p* = 0.086. The classification × position interaction was not significant, *F*_(6, 258)_ = 0.81, *p* = 0.560, indicating that the association between classification and mental toughness was consistent across positional roles rather than confined to any particular position (Table 6).

**TABLE 6:** Two-way ANOVA of mental toughness by 2D:4D-derived classification and playing position.

Source	df	F	*p*
Classification (Type A / B / C)	2	6.10	0.003
Playing position	3	2.22	0.086
Classification × Playing position	6	0.81	0.560
Error	258	–	–

Statistical significance was set at *p* < 0.05.

Because the analytical treatment of biological sex requires transparency, the sex difference was also examined directly rather than left implicit. Male players reported higher mental toughness (*M* = 44.90, SD = 6.05) than female players (*M* = 42.48, SD = 7.11), t(268) = 1.99, *p* = 0.047. This difference was not attenuated when 2D:4D-derived classification was taken into account: in a model containing both factors, the effect of sex was larger, *F*_(1, 266)_ = 8.06, *p* = 0.005, alongside the effect of classification, *F*_(2, 266)_ = 8.80, *p* < 0.001. The two factors act in opposing directions in this sample, which explains the pattern. Female players were substantially over-represented in Type B (41.4% of women, compared with 17.0% of men), the category with the highest mean mental toughness, so the unadjusted sex difference understates the adjusted one. These estimates rest on 29 female players and are reported for transparency rather than as a basis for inference about sex differences in mental toughness; the implications of the imbalance are set out in the Limitations.

## 4 Discussion

The present study examined whether mental toughness in amateur soccer players varies with 2D:4D-derived classification and playing position. Two main findings emerged: players assigned to Type B reported higher mental toughness than Type A and Type C players, and defenders reported higher mental toughness than goalkeepers. Interpreted within a neuropsychological framework, these results are consistent with the view that different neural profiles support different task demands, but they should be read as associations rather than as evidence of categorical, sex-linked brain differences.

Classical work indicates that hemispheres can be differentially engaged depending on task demands (Sperry, 1975; Iaccino, 2014), and soccer-specific creativity has been shown to recruit a distributed network involved in semantic processing, visual and motor imagery, and sensorimotor integration (Fink et al., 2019). Because soccer is inherently a multitasking game, a player’s cognitive profile, and its fit with positional demands, may plausibly contribute to mental toughness. At the same time, contemporary large-scale evidence cautions against equating such profiles with a binary “male/female brain”: most individuals present a mosaic of so-called masculine and feminine features (Joel et al., 2015), and meta-analytic syntheses report few reliable male–female differences beyond overall size (Eliot et al., 2021). The Type A, Type B and Type C labels used here should therefore be read strictly as operational descriptors of 2D:4D-linked variation, and not as claims about categorically sexed brains.

Our findings align with a broader literature relating 2D:4D to soccer-relevant traits (Perciavalle et al., 2013; Mailhos et al., 2013, 2016; Romann and Fuchslocher, 2015; Bilgiç et al., 2016; Acar and Tutkun, 2019; Islam and Kundu, 2019, 2020; Nobari et al., 2021). Perciavalle et al. (2013) reported that players with lower 2D:4D ratios show more aggression, and Mailhos et al. (2016) found that players receiving red cards had more masculinized (lower) 2D:4D ratios, suggesting that a more androgenized (lower) digit-ratio profile may be associated with greater on-field aggression. Romann and Fuchslocher (2015) proposed 2D:4D as a supplementary criterion in youth talent evaluation, whereas Bilgiç et al. (2016) found no association between 2D:4D and sporting performance across several sports. Taken together, these mixed results suggest that 2D:4D may be more informative about psychological and behavioral tendencies than about raw physical performance, which is consistent with our observation that the less androgenized Type B profile was associated with higher mental toughness. Acar and Tutkun (2019) likewise reported that 2D:4D discriminated national from amateur footballers, while cautioning that it should be combined with other talent-identification parameters.

Islam and Kundu (2019) suggested that higher prenatal testosterone may enhance soccer performance and cognitive ability. Our results qualify this claim: male participants assigned to Type B showed greater mental toughness than players assigned to Type A, indicating that the 2D:4D-derived classification carries information about mental toughness that is not reducible to sex category, although, as reported in the Results, a sex difference was also present in these data and the two factors operate in opposing directions. This is consistent with the view that the prenatal androgen environment varies continuously both within and between the sexes, so that an individual profile need not follow from sex category (Manning et al., 2000; Karaismailoglu, 2020), and with evidence that global functional connectivity shows age- and sex-related shifts across the lifespan (Zhang et al., 2021). Accordingly, our data do not support the assumption that men are inherently more mentally tough than women; instead, they point to prenatally influenced neurocognitive variation as the more relevant factor (Canan, 2019).

Considered specifically in terms of sex-related variation in cognition and behavior, the present pattern is informative in two respects. First, the digit-ratio index and sex category carry partly distinct information. Players of both sexes were distributed across all three categories, and the classification effect persisted when sex was included in the model, just as the sex effect persisted when classification was included. Notably, the two run counter to one another here: female players were over-represented in the highest-scoring category yet scored lower overall, so neither factor is reducible to the other, and a study reporting only one of them would have given a misleading account. This is consistent with mosaic accounts of sex-related neural variation, in which within-sex variability substantially exceeds the average between-sex difference and individuals present combinations of features rather than a coherent male or female configuration (Ingalhalikar et al., 2014; Joel et al., 2015; Eliot et al., 2021; Zhang et al., 2021). Second, the direction of the effect runs counter to a simple masculinization account. Had prenatal androgenization straightforwardly conferred psychological robustness, the more androgenized Type A profile should have shown the highest mental toughness; the opposite was observed. This argues against treating mental toughness as a masculine trait and suggests that the relationship between the prenatal hormonal milieu and the adult psychological phenotype is neither linear nor uniformly directed toward greater masculinization. More generally, the findings illustrate why sex-related biological variation is better modelled as a continuum than as a dichotomy: a straightforward comparison of men and women in this sample would have obscured a difference that a graded, prenatally influenced index was able to detect. Because effect sizes throughout the 2D:4D literature are typically small, and because the present classification rests on a single anthropometric index, these implications are offered as interpretive rather than confirmatory, and they indicate a direction for adequately powered, sex-balanced replication rather than a settled conclusion.

With respect to position, goalkeepers reported lower mental toughness than defenders despite the distinctive pressures of their role. This partly contrasts with Asamoah and Grobbelaar (2016), who found that goalkeepers and forward scored higher than midfielders, and with Yazıcı et al. (2021), who reported no significant positional differences. The small goalkeeper subsample in our study (*n* = 23) limits the reliability of this comparison, and the finding should be regarded as preliminary and interpreted cautiously.

### 4.1 Limitations of the study

Several limitations should be considered when interpreting the findings of this study. First, the cross-sectional design precludes causal inference, and the observed associations may have been influenced by unmeasured confounding factors, such as training history, competitive level, or individual motivation. Second, the tripartite classification based on the 2D:4D ratio remains a debated heuristic, since the ratio is only a weak and indirect proxy for prenatal androgen exposure; Types A, B and C are therefore operational categories rather than validated neural phenotypes, and although the classification threshold is defined explicitly in the Methods, it has not been externally validated. In addition, although digit lengths were obtained under a standardized protocol with a calibrated digital caliper, direct manual measurement remains more susceptible to observer-related error than computer-assisted analysis of hand scans (Allaway et al., 2009), and formal inter- and intra-rater reliability coefficients were not computed. Mental toughness was evaluated using only the Sports Mental Toughness Questionnaire (SMTQ), making the findings vulnerable to social desirability and common-method bias; incorporating objective or multi-method assessments would strengthen construct validity. Furthermore, the sample consisted of 270 amateur football players from a single region of Türkiye, with an uneven sex distribution (10.7% women) and a relatively small goalkeeper subgroup (*n* = 23), which limits the generalizability of the findings and reduces statistical power for subgroup comparisons. Finally, reporting comprehensive effect size estimates together with complete omnibus ANOVA statistics would improve the transparency and interpretability of the statistical analyses.

Three further limitations should be noted. First, the sample consisted entirely of amateur players, which restricts generalization to professional athletes and to other sports. Second, the goalkeeper subgroup (*n* = 23) was small relative to the other positions, which reduced the statistical power available for position-specific comparisons and means that the goalkeeper–defender contrast should be treated as preliminary. Third, mental toughness was assessed with a single validated instrument, so the findings reflect the construct as operationalized by the SMTQ and may not capture every facet of mental toughness.

A further limitation follows from the analytical treatment of biological sex described in the Methods. Because the sex distribution of the sample was strongly unbalanced and because sex and 2D:4D-derived classification are not statistically independent, sex could not be entered as an inferential factor. The present design therefore cannot establish whether the association between classification and mental toughness is invariant across sexes, and the sex difference reported in the Results, although statistically significant, rests on only 29 female players and should be regarded as descriptive. Replication in sex-balanced samples is required before the findings are generalized to female players specifically.

A further limitation concerns the age range of the sample. Players spanned the 13–17, 18–21, 22–27 and 28-and-over bands, and approximately one third were below 18 years of age. Although the 2D:4D ratio is prenatally established and is not expected to change with subsequent growth, psychological maturation across adolescence and early adulthood may itself contribute to variation in mental toughness, and the present design cannot separate that contribution from the associations reported here. Age-stratified replication is therefore desirable.

### 4.2 Future directions

Despite these limitations, the findings provide several practical implications and directions for future research. Players’ 2D:4D profiles may be considered as a complementary characteristic, alongside established physiological, technical, and psychological assessments, when individualizing athlete evaluation and training programs. Likewise, training interventions may be adapted according to positional demands and cognitive characteristics to better support performance development. Future studies should include larger and more balanced samples across competitive levels, playing positions, and both sexes while pre-registering classification thresholds and statistical analysis plans to enhance methodological rigor and reproducibility. In addition, the use of automated image-based or wearable technologies for 2D:4D assessment, together with objective in-game cognitive and behavioral performance indicators, may improve measurement precision, ecological validity, and the practical applicability of future research.

Three specific directions follow from the analyses reported here. First, adequately powered multifactorial designs are needed: the present sample supported a two-factor model, but a three-factor model additionally incorporating training experience produced cells too sparse for stable estimation, and larger samples would allow the joint contribution of classification, position and experience–including their higher-order interactions–to be evaluated. Second, inter- and intra-rater reliability should be reported routinely, and computer-assisted measurement of hand scans should be preferred where feasible. Third, prospective and longitudinal designs, combined with pre-registered classification thresholds, would allow the stability and predictive value of the classification to be assessed rather than assumed.

## 5 Conclusion

In amateur soccer players, mental toughness varied with the 2D:4D-derived classification and with playing position: players assigned to Type B, and defenders, reported the highest mental toughness. A difference by biological sex was also present, with male players scoring higher, but the two effects ran in opposing directions and neither proved reducible to the other. Because 2D:4D indexes the prenatal androgen environment, this pattern indicates that the relevant sex-related variation in this trait is continuous and graded rather than organized around the male–female distinction, and that its direction is not one of increasing masculinization. These findings suggest that neuropsychological indicators may usefully complement, but not replace, traditional talent-identification criteria, and that coaches seeking specific attributes such as creativity or mental toughness might consider cognitive profile when assigning positions. As Johan Cruyff observed, “football is a game you play with your brain.” At the same time, Types A, B and C remain operational classifications derived from a single anthropometric index rather than literal brain categories, and given the contested status of binary models of brain organization, these conclusions should be treated as hypotheses to be tested in larger, sex-balanced, methodologically rigorous and pre-registered studies.

## Data Availability

The raw data supporting the conclusions of this article will be made available by the authors, without undue reservation.

## References

[B1] Acar H. Tutkun E. (2019). Analysis of the 2D: 4D ratios of national and amateur football players. *Int. J. Appl. Exerc. Physiol.* 8 132–137. 10.30472/IJAEP.V8I1.326

[B2] Allaway H. Bloski T. Pierson R. Lujan M. (2009). Digit ratios (2D:4D) determined by computer-assisted analysis are more reliable than those using physical measurements, photocopies, and printed scans. *Am. J. Hum. Biol.* 21 365–370. 10.1002/ajhb.20892 19263413 PMC2882434

[B3] Altıntaş A. Koruç P. B. (2016). Sporda zihinsel dayanıklılık envanteri’nin psikometrik özelliklerinin incelenmesi (SZDE). *Spor Bilimleri Dergisi* 27 163–171. Turkish. 10.17644/sbd.311985

[B4] Asamoah B. Grobbelaar H. W. (2016). Positional comparisons of mental toughness, psychological skills and group cohesion among soccer players psychology. *African J. Phys. Activity Health Sci.* 22 747–759.

[B5] Auger J. Le Denmat D. Berges R. Doridot L. Salmon B. Canivenc-Lavier M. et al. (2013). Environmental levels of oestrogenic and antiandrogenic compounds feminize digit ratios in male rats and their unexposed male progeny. *Proc. Biol. Sci.* 280:20131532. 10.1098/rspb.2013.1532 23926155 PMC3757980

[B6] Baker F. (1888). Anthropological notes on the human hand. *Am. Anthropol.* 1 51–76. 10.1525/aa.1888.1.1.02a00040

[B7] Berenbaum S. Bryk K. Nowak N. Quigley C. Moffat S. (2009). Fingers as a marker of prenatal androgen exposure. *Endocrinology* 150 5119–5124. 10.1210/en.2009-0774 19819951 PMC2775980

[B8] Bilgiç M. Biçer M. Özdal M. (2016). Farklı branşlarda spor yapan 11-13 yaşlarda grubu çocukların 2D: 4D parmak oranlarının sportif performansla ilişkisinin incelenmesi. *Gaziantep Üniver. Spor Bilimleri Dergisi* 1 48–56. Available online at: https://izlik.org/JA52ET53DD Turkish

[B9] Bloomfield J. Polman R. O’Donoghue P. (2007). Physical demands of different positions in FA premier league soccer. *J. Sports Sci. Med.* 6 63–70. 24149226 PMC3778701

[B10] Brown W. Finn C. Breedlove S. (2002). Sexual dimorphism in digit-length ratios of laboratory mice. *Anat. Rec.* 267 231–234. 10.1002/ar.10108 12115273

[B11] Bull S. J. Shambrook C. J. James W. Brooks J. E. (2005). Towards an understanding of mental toughness in elite English cricketers. *J. Appl. Sport Psychol.* 17 209–227. 10.1080/10413200591010085

[B12] Canan S. (2019). *İFA İnsanın Fabrika Ayarları-1: Beden (Tuti Kitap).* Tuti Kitap. Turkish.

[B13] Carling C. Williams A. M. Reilly T. (2007). *Handbook of Soccer Match Analysis: A Systematic Approach to Improving Performance.* Milton Park: Routledge.

[B14] Clough P. Earle K. Sewell D. (2002). “Mental toughness: The concept and its measurement,” in *Solutions in Sport Psychology*, ed. Cockerill I. (Boston, MA: Thomson Learning).

[B15] Cohen J. (1988). *Statistical Power Analysis for the Behavioral Sciences*. Florence: Taylor & Francis Group.

[B16] Cohen J. (2013). *Statistical Power Analysis for the Behavioral Sciences.* Milton Park: Routledge.

[B17] Crust L. Clough P. J. (2011). Developing mental toughness: From research to practice. *J. Sport Psychol. Action* 2 21–32. 10.1080/21520704.2011.563436

[B18] Davidshofer K. R. Murphy C. O. (2005). *Psychological Testing: Principles and Applications.* Upper Saddle River, NJ: Prentice Hall.

[B19] Diment G. M. (2014). Mental skills training in soccer: A drill-based approach. *J. Sport Psychol. Action* 5 14–27. 10.1080/21520704.2013.865005

[B20] Eklund E. Ekström L. Thörngren J. Ericsson M. Berglund B. Hirschberg A. (2020). Digit ratio (2D:4D) and Physical performance in female olympic athletes. *Front. Endocrinol.* 11:292. 10.3389/fendo.2020.00292 32528408 PMC7247813

[B21] Eliot L. Ahmed A. Khan H. Patel J. (2021). Dump the dimorphism: Comprehensive synthesis of human brain studies reveals few male-female differences beyond size. *Neurosci. Biobehav. Rev.* 125 667–697. 10.1016/j.neubiorev.2021.02.026 33621637

[B22] Faul F. Erdfelder E. Lang A. Buchner A. (2007). G*Power 3: A flexible statistical power analysis program for the social, behavioral, and biomedical sciences. *Behav. Res. Methods* 39 175–191. 10.3758/bf03193146 17695343

[B23] Fink A. Bay J. Koschutnig K. Prettenthaler K. Rominger C. Benedek M. et al. (2019). Brain and soccer: Functional patterns of brain activity during the generation of creative moves in real soccer decision-making situations. *Hum. Brain Mapp.* 40 755–764. 10.1002/hbm.24408 30259600 PMC6492000

[B24] George D. Mallery P. (2016). *IBM SPSS Statistics 23 Step by Step: A Simple Guide and Reference*, 14th Edn. Milton Park: Routledge, 10.4324/9781315545899

[B25] George R. (1930). Human finger types. *Anatomical Rec.* 46 199–204. 10.1002/ar.1090460210

[B26] Gucciardi D. Hanton S. Fleming S. (2017). Are mental toughness and mental health contradictory concepts in elite sport? A narrative review of theory and evidence. *J. Sci. Med. Sport* 20 307–311. 10.1016/j.jsams.2016.08.006 27568074

[B27] Hampson E. Ellis C. Tenk C. (2008). On the relation between 2D:4D and sex-dimorphic personality traits. *Arch. Sex Behav.* 37 133–144. 10.1007/s10508-007-9263-3 18075733

[B28] Hönekopp J. Watson S. (2010). Meta-analysis of digit ratio 2D:4D shows greater sex difference in the right hand. *Am. J. Hum. Biol.* 22 619–630. 10.1002/ajhb.21054 20737609

[B29] Iaccino J. F. (2014). *Left Brain-Right Brain Differences: Inquiries, Evidence, and New Approaches.* London: Psychology Press.

[B30] Ingalhalikar M. Smith A. Parker D. Satterthwaite T. Elliott M. Ruparel K. et al. (2014). Sex differences in the structural connectome of the human brain. *Proc. Natl. Acad. Sci. U S A.* 111 823–828. 10.1073/pnas.1316909110 24297904 PMC3896179

[B31] Islam M. S. Kundu B. (2019). Digit ratio and soccer. *Orthopedics Sports Med. Open Access J.* 3 227–230. 10.32474/OSMOAJ.2019.03.000154

[B32] Islam M. S. Kundu B. (2020). Soccer passing accuracy differentiates between high and low digit ratio (2D:4D) soccer players. *Am. J. Sports Sci.* 8 49–55. 10.11648/j.ajss.20200802.13

[B33] Joel D. Berman Z. Tavor I. Wexler N. Gaber O. Stein Y. et al. (2015). Sex beyond the genitalia: The human brain mosaic. *Proc. Natl. Acad. Sci. U S A.* 112 15468–15473. 10.1073/pnas.1509654112 26621705 PMC4687544

[B34] Jones G. Hanton S. Connaughton D. A. (2007). framework of mental toughness in the world’s best performers. *Sport Psychol.* 21 243–264. 10.1123/tsp.21.2.243

[B35] Karaismailoglu S. (2020). *Kadın beyni, erkek beyni*, 14th Edn. Ankara: Elma Yayınevi. Turkish.

[B36] Kashima Y. (2000). Conceptions of culture and person for psychology. *J. Cross-Cult. Psychol.* 31 14–32. 10.1177/0022022100031001003

[B37] Lustig C. Shah P. Seidler R. Reuter-Lorenz P. (2009). Aging, training, and the brain: A review and future directions. *Neuropsychol. Rev.* 19 504–522. 10.1007/s11065-009-9119-9 19876740 PMC3005345

[B38] Mailhos A. Buunk A. P. del Arca D. Tutte V. (2013). Mild positive correlation of the 2D:4D ratio with aggressive dominance, but not with sociable dominance, in junior soccer players. *Pers. Individ. Dif.* 55 703–707.

[B39] Mailhos A. Buunk A. Del Arca D. Tutte V. (2016). Soccer players awarded one or more red cards exhibit lower 2D:4D ratios. *Aggress. Behav.* 42 417–426. 10.1002/ab.21638 26699684

[B40] Manning J. T. Trivers R. Fink B. (2017). Is digit ratio (2D:4D) related to masculinity and femininity? Evidence from the BBC internet study. *Evol. Psychol. Sci.* 3 316–324. 10.1007/s40806-017-0098-4

[B41] Manning J. Barley L. Walton J. Lewis-Jones D. Trivers R. Singh D. et al. (2000). The 2nd:4th digit ratio, sexual dimorphism, population differences, and reproductive success. Evidence for sexually antagonistic genes? *Evol. Hum. Behav.* 21 163–183. 10.1016/s1090-5138(00)00029-5 10828555

[B42] Manning J. Kilduff L. Cook C. Crewther B. Fink B. (2014). Digit ratio (2D:4D): A biomarker for prenatal sex steroids and adult sex steroids in challenge situations. *Front. Endocrinol.* 5:9. 10.3389/fendo.2014.00009 24523714 PMC3906590

[B43] Manno F. (2008). Measurement of the digit lengths and the anogenital distance in mice. *Physiol. Behav.* 93 364–368. 10.1016/j.physbeh.2007.09.011 18054055

[B44] Nobari H. Alves A. Clemente F. Pérez-Gómez J. (2021). Influence of 2D:4D ratio on fitness parameters and accumulated training load in elite youth soccer players. *BMC Sports Sci. Med. Rehabil.* 13:125. 10.1186/s13102-021-00354-5 34635156 PMC8504125

[B45] Perciavalle V. Di Corrado D. Petralia M. Gurrisi L. Massimino S. Coco M. (2013). The second-to-fourth digit ratio correlates with aggressive behavior in professional soccer players. *Mol. Med. Rep.* 7 1733–1738. 10.3892/mmr.2013.1426 23588344 PMC3694562

[B46] Phelps V. R. (1952). Relative index finger length as a sex-influenced trait in man. *Am. J. Hum. Genet.* 4 72–89. 14943709 PMC1716436

[B47] Romann M. Fuchslocher J. (2015). Validation of digit-length ratio (2D:4D) assessments on the basis of DXA-derived hand scans. *BMC Med. Imaging* 15:1. 10.1186/s12880-015-0042-7 25645550 PMC4323124

[B48] Schumacher N. Schmidt M. Wellmann K. Braumann K. (2018). General perceptual-cognitive abilities: Age and position in soccer. *PLoS One* 13:e0202627. 10.1371/journal.pone.0202627 30138420 PMC6107215

[B49] Sheard M. Golby J. van Wersch A. (2009). Progress toward construct validation of the Sports mental toughness questionnaire (SMTQ). *Eur. J. Psychol. Assess.* 25 186–193. 10.1027/1015-5759.25.3.186

[B50] Sperry R. W. (1975). Left-brain, right-brain. *Saturday Rev.* 2 30–32.

[B51] Surujlal J. (2016). Influence of organizational support on retirement planning and financial management of professional soccer players. *Polish J. Manag. Stud.* 13 164–174. 10.17512/pjms.2016.13.2.16

[B52] van Honk J. Schutter D. Bos P. Kruijt A. Lentjes E. Baron-Cohen S. (2011). Testosterone administration impairs cognitive empathy in women depending on second-to-fourth digit ratio. *Proc. Natl. Acad. Sci. U S A.* 108 3448–3452. 10.1073/pnas.1011891108 21300863 PMC3044405

[B53] Vaughan R. Hanna D. Breslin G. (2018). Psychometric properties of the Mental toughness questionnaire 48 (MTQ48) in elite, amateur and nonathletes. *Sport Exerc. Performance Psychol.* 7 128–140. 10.1037/spy0000114

[B54] Williams A. Ford P. Drust B. (2020). Talent identification and development in soccer since the millennium. *J. Sports Sci.* 38 1199–1210. 10.1080/02640414.2020.1766647 32568000

[B55] Yazıcı A. Yapar A. Güven S. Taşcıoğlu R. Ulutaş I. (2021). Comparison of emotional intelligence and mental toughness of youth soccer players in terms of position and soccer experience variables. *Gazi Beden Eğitimi ve Spor Bilimleri Dergisi* 26 407–418. 10.53434/gbesbd.938664

[B56] Zhang Y. Luo Q. Huang C. Lo C. Langley C. Desrivières S. et al. (2021). The human brain is best described as being on a female/male continuum: Evidence from a neuroimaging connectivity study. *Cereb. Cortex* 31 3021–3033. 10.1093/cercor/bhaa408 33471126 PMC8107794

[B57] Zhao D. Yu K. Zhang X. Zheng L. (2013). A comparison of measurement methods and sexual dimorphism for digit ratio (2D:4D) in Han ethnicity. *Arch. Sexual Behav.* 42 1899–1906. 10.1007/s10508-013-0111-3 24013635 PMC3890058

